# Development and optimization of mathematical models for uniform seed placement in precision black cumin seeding under laboratory conditions

**DOI:** 10.7717/peerj.20755

**Published:** 2026-02-04

**Authors:** Gulin Turkusay, Arzu Yazgi

**Affiliations:** 1 Dept. of Agricultural Engineering and Technologies/Graduate School of Natural and Applied Sciences, Ege University, Izmir, Turkey; 2 Dept. of Agricultural Engineering and Technologies/Faculty of Agriculture, Ege University, Izmir, Turkey

**Keywords:** Flow accuracy, Medicinal and aromatic plants, Metering device, Microgranular applicator, Response surface methodology

## Abstract

**Objectives:**

The objective of the study was to quantify seed flow consistency and in-row spacing accuracy when using a conveyor belt-style metering device under varying operational parameters, to develop mathematical models, and to optimize the uniformity of seed placement for black cumin seeding.

**Methods:**

Seed flow properties and the uniformity of in-row seed distribution uniformity were evaluated through weighing tests and sticky belt methods, respectively. The uniformity of the flow was assessed using coefficient of variation (CV) values, while the in-row seed distribution uniformity was evaluated by using the variation factor (V_f_) and the goodness criterion (*λ*). The experiments were conducted based on Central Composite Design (CCD) under laboratory conditions. The forward speed, seed rate, and seed falling angle were chosen as independent variables. The experiments were conducted at five levels of forward speed (1.01, 1.4, 2.0, 2.6, and 3.01 m s^−1^), five levels of seed rate (6.6, 10, 15, 20 and 23.4 kg ha^−1^), and five levels of seed falling angle (1.36, 15, 35, 55 and 68.64°).

**Results:**

The polynomial functions were developed and the V_f_ and *λ* models were optimized. Optimization reduced the variation factor (V_f_) to 0.53 and improved the goodness criterion (*λ*) to 91.67%, indicating a substantial enhancement in seed placement uniformity. For the V_f_ model, the optimum forward speed was found to be 1.05 m s^−1^, with a seed rate of 12.35 kg ha^−1^ and a seed falling angle of 35°; whereas for the *λ* model, the corresponding values were calculated 1.55 m s^−1^, 21.1 kg ha^−1^, and 28.5°, respectively. The developed models showed high predictive accuracy, with an *R*^2^ value of 95.96% for the *λ* model. Based on the findings of this work, the seed rate was determined as the most important parameter for all models considered.

**Conclusions:**

The results of the experiments also revealed that the conveyor belt metering unit could be used for the seeding process of black cumin seeds with great success, without encountering significant problems. The comparison of *R*^2^ value of the *λ* and V_f_ models indicated that the *λ* model had a better variable prediction. Therefore, the optimum values from the *λ* model may be more useful than those from the V_f_ model.

## Introduction

Medicinal and aromatic plants have been integral to a wide range of sectors—including medicine, food technology, the chemical industry, cosmetics, and beverage production—and their global demand has shown a continuous upward trend. Countries such as China, India, the United States, Germany, Mexico, Egypt, Bulgaria, Chile, Singapore, Morocco, Pakistan, and Turkey are among the leading actors in the international trade of these plant groups, serving as key importers and exporters (Boztaş et al., 2021). With the rising interest in these products, the cultivation of medicinal and aromatic species has become increasingly important. Improvements made at any point in the cultivation chain, particularly during planting operations, can markedly enhance both the quality and the yield of the harvested material, creating significant added value for producers and end products alike.

Research on medicinal and aromatic plants has predominantly addressed several key themes. These include investigations into how climatic conditions influence plant growth (Soleymani, Amirnia & Ghiyasi, 2016), studies aimed at identifying alternative utilization areas for these species (Aytaç & Yiğen, 2016), and work focusing on appropriate harvesting and drying techniques (Bayram et al., 2010; Kırpık & Özgüven, 2018; Singh & Malhotra, 2007; Inan, 2019; Koşar & Özel, 2018). In addition, numerous studies have examined the optimization of essential oil or mineral extraction processes from these plants (Bostan, Ravazi & Farhoosh, 2010; Mitić et al., 2019; Hosseini & Razavi, 2020; Durovic et al., 2022).

Research on the mechanization of planting, harvesting, and drying has predominantly been performed by agronomists, whereas studies focusing on mathematical modeling and the optimization of oil or mineral extraction processes have generally been undertaken by chemists. In these chemistry-oriented investigations, scholars have examined how various operational parameters influence the extraction of oils and minerals from medicinal and aromatic plants, aiming to identify the key factors governing these processes and to determine the optimal extraction conditions (Bostan, Ravazi & Farhoosh, 2010; Mitić et al., 2019; Hosseini & Razavi, 2020; Durovic et al., 2022).

In contrast, studies carried out by agronomists have generally relied on manual seeding, with subsequent evaluations centered on plant yield and quality characteristics (Veselinov et al., 2014; Khorie et al., 2015; Sönmez et al., 2019). Only a limited number of investigations have integrated the use of mechanical seeders while also assessing machine performance, developing mathematical models, and optimizing seeding parameters for these plant species (Özdemir & Degirmencioglu, 2020; Varol & Degirmencioglu, 2022; Turkusay & Yazgi, 2024).

Özdemir & Degirmencioglu (2020) conducted a study to develop a mathematical model capable of predicting the volumetric efficiency of fluted rollers when metering different crop seeds. Their research incorporated barley, alfalfa, flax, coriander, safflower, oat, sesame, wheat, and rye seeds, and five separate volumetric efficiency models were generated. A noteworthy result of the study was the observation that the coefficient of variation (CV) values—used to indicate the uniformity of seed flow—were substantially lower than those reported in earlier publications. Among the 332 experimental runs, CV values were predominantly found in the ranges of CV < 1 (78%) and 1 < CV < 2 (17.8%). When all replications were considered, 99.7% of the CV measurements fell within CV < 4.

In their research, Varol & Degirmencioglu (2022) assessed the performance of a vacuum-based precision metering unit used for coriander seed placement. The study examined how the diameter of the holes on the vacuum plate, the forward operating speed, and the magnitude of the vacuum pressure influenced seed spacing accuracy. A polynomial equation was constructed to represent the system behavior and was later optimized. The optimal operating parameters derived from this model were identified as a hole diameter of 1.75 mm, a forward speed of 1.5 m s^−^^1^, and a vacuum pressure of 47.7 mbar.

Turkusay & Yazgi (2024) examined how various operational factors influence seed flow accuracy and in-row distribution uniformity using a micro-granular applicator. In their study, mathematical models were developed and optimized to characterize the behavior of the system, and the results showed that forward speed had the strongest impact on flow consistency. Both V_f_ and*λ* models were formulated and refined through optimization. The goodness criterion increased markedly from 58.00% to 89% after optimization, whereas the variation factor improved by decreasing from 1.53 to 0.71.

In another study conducted by Turkusay & Yazgi (2025), the potential of a conveyor belt-type metering device for precision coriander seeding was examined by assessing seed flow behavior and in-row spacing performance under various operating conditions. Polynomial functions were developed for both V_f_ and *λ* responses, and optimization of these models identified the most suitable operating parameters. For the V_f_model, the optimal conditions were a forward speed of 1.67 m s^−^^1^, a seed rate of 14.7 kg ha^−^^1^, and a seed falling angle of 25.9°, while the *λ* model yielded optimal values of 1.68 m s^−^^1^, 20.4 kg ha^−^^1^, and 31.5°. Among all evaluated factors, seed rate was found to be the most influential.

A shared characteristic of the seeding studies conducted with coriander seeds (Özdemir & Degirmencioglu, 2020; Varol & Degirmencioglu, 2022) was that each employed a different type of metering mechanism. In contrast, studies conducted by similar seed metering device using sage and coriander seeds and focused on generating results that were specific to the properties of that seed types (Turkusay & Yazgi, 2024; Turkusay & Yazgi, 2025).

In contrast to earlier studies, the main objective of this research was to examine the feasibility of seeding black cumin seeds by a conveyor belt-type metering unit that enables precise adjustment of seed rate, while also assessing seed flow accuracy and enhancing the uniformity of in-row seed placement. To achieve this aim, a range of operational settings was tested. In addition, polynomial equations were formulated to determine the optimal conditions appropriate for specifically black cumin seeds.

## Materials & Methods

**Figure 1:**
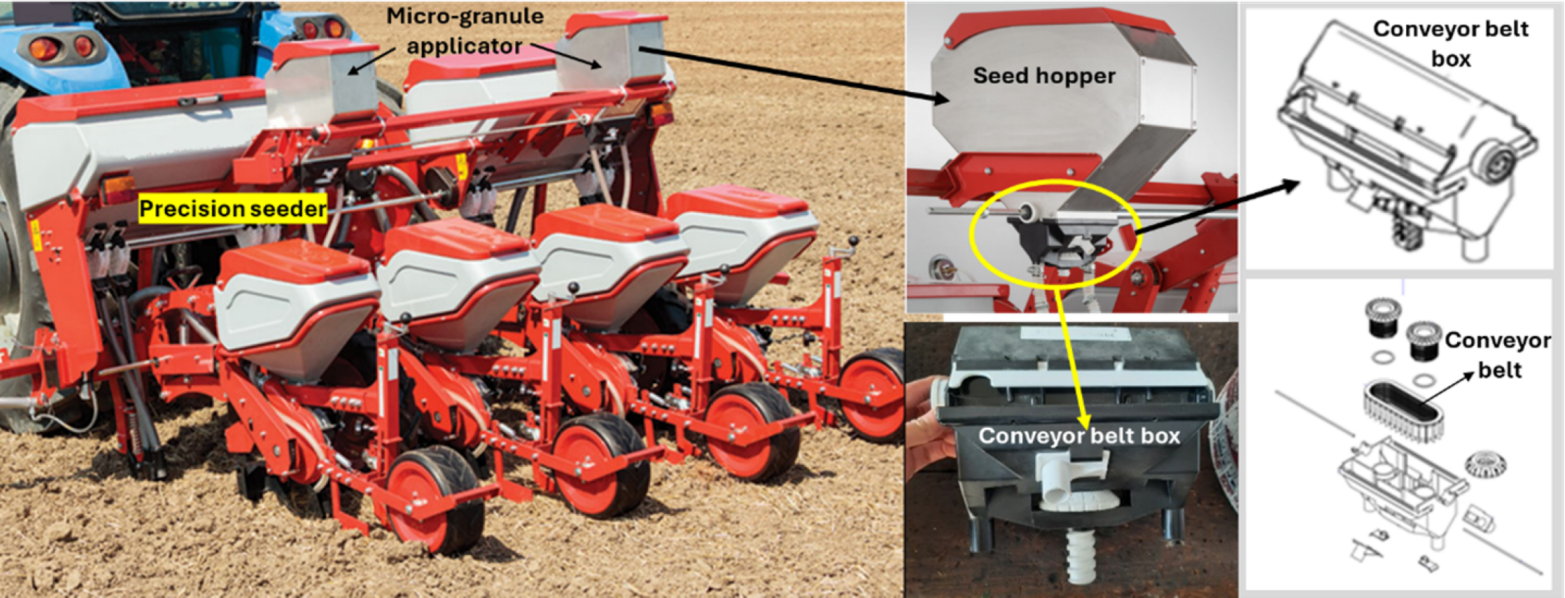
Conveyor belt type seed metering unit (adapted from the MaterMacc, 2021).

A conveyor belt type seed metering unit, originally designed as a micro-granular applicator, was employed in the experiments as depicted in Fig. 1. This mechanism was preferred because the physical characteristics of the black cumin seeds (such as small size, irregular shape, and low flowability) make it difficult to achieve stable flow using conventional systems like fluted rollers. The seed metering unit with two different seed outlets is positioned under the seed hopper and is equipped with a mechanism that enables easy adjustment to the desired seed rate values. Some of the physical properties of the black cumin seeds used in this work are displayed in Table 1.

**Table 1:** Some physical properties of the black cumin seeds.

Physical property	Average^*^
Length, l (mm)	2.79 (0.31)
Width, w (mm)	1.53 (0.16)
Thickness, t, (mm)	1.16 (0.19)
Sphericity, Φ (%)	61.16 (6.17)
Thousand seed mass, m_1000_ (g)	3.08 (0.01)

**Notes.**

* The numbers in the brackets indicate the standard deviation.

**Figure 2:**
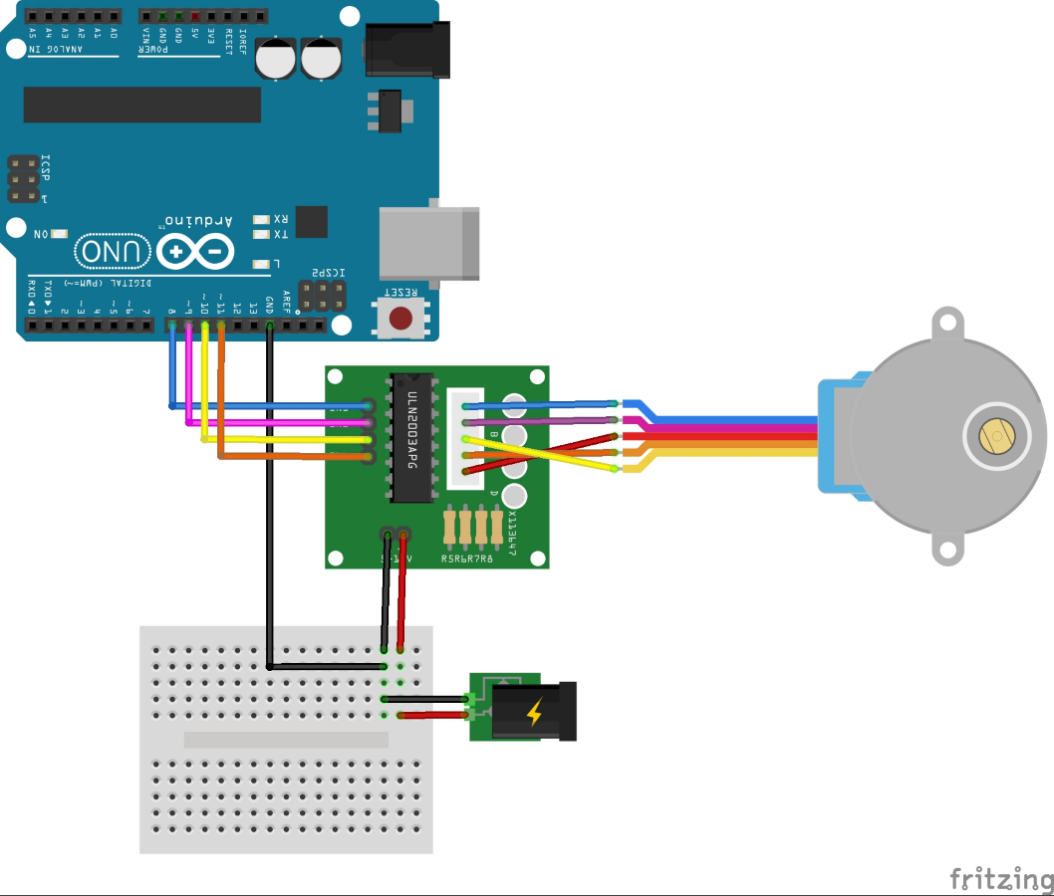
The schematic diagram of the Arduino circuits.

The experiments were conducted in laboratory conditions by utilizing a test stand similar to that used by Turkusay & Yazgi (2024) and Turkusay & Yazgi (2025). Data were also collected as previously described in Turkusay & Yazgi (2024) and Turkusay & Yazgi (2025). For the experiments, a test stand was assembled that included a seeding unit, a carrier frame for seeding unit, a stepper motor, a stepper motor control unit, a stepper motor control driver, a power supply, a data cable and a computer. The conveyor belt was powered by a computer-controlled stepper motor. The control setup for the stepper motor included an Arduino Uno R3, an Arduino CNC Shield, a DRV8825 driver module, a DC power adapter in a 19 V and 4.74 A, a communication cable, and a Nema 23 stepper motor with a torque rating of 0.55 Nm. The schematic diagram of the Arduino circuits is provided in Fig. 2. The seeding unit was installed on the carrier frame through a continuously adjustable mounting height.

**Figure 3:**
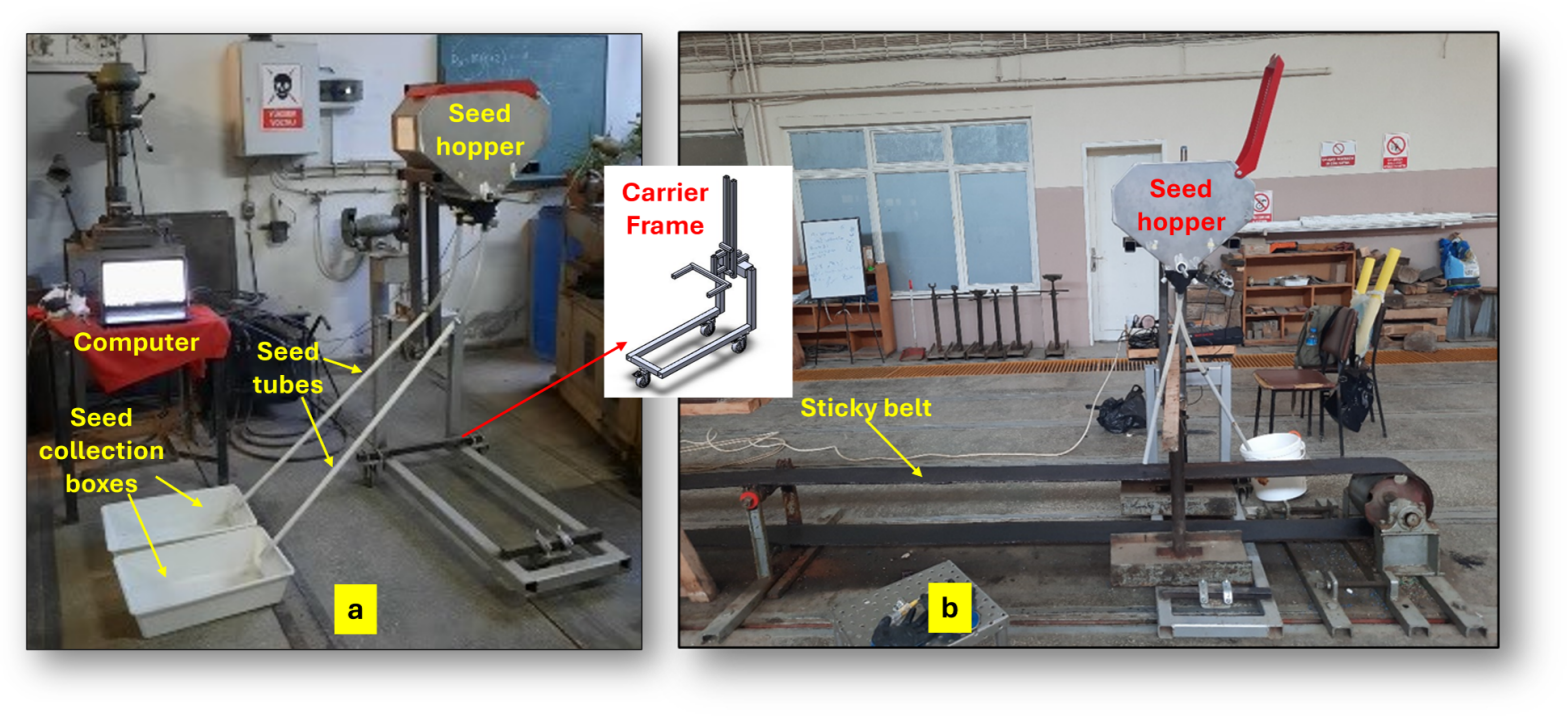
Test stands specifically designed for performing weighing (A) and sticky belt (B) experiments.

In this study, the performance of the conveyor belt-type seed metering device was evaluated with respect to seed rate, flow uniformity, and in-row seed distribution uniformity based on International Organization of Standards (1984) and Önal (2017) and Griepentrog (1994). Seed rate and flow characteristics were assessed through weighing experiments, whereas in-row seed distribution uniformity was evaluated using sticky belt tests. Figure 3 illustrates the experimental arrangements for the weighing tests (Fig. 3A) and the sticky belt tests (Fig. 3B). During all experiments, the seed metering system was synchronized with the operating speed of the sticky belt to ensure coordinated movement. The rotational speed of the metering unit corresponding to the forward speed of the sticky belt was calculated and entered to the program interface, enabling the stepper motor to operate at the desired rotational speed.

**Figure 4:**
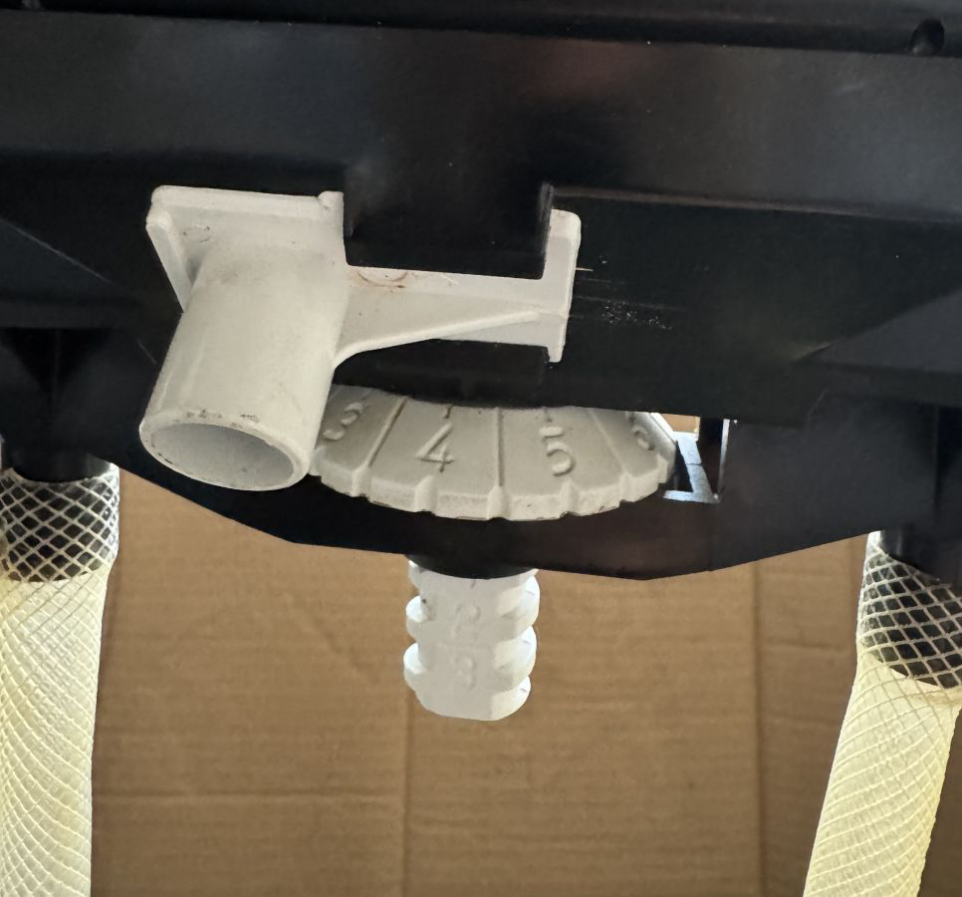
Seed rate-adjustment mechanism.

To identify the seed rate for black cumin, weighing trials were conducted at different adjustment levels and positions of the conveyor belt-type metering device. As shown in Fig. 4, the seed rate control system provided 20 possible scale combinations, with horizontal settings between 0 and 9.5 and vertical settings between 0 and 3 at each position. The system incorporated a precise adjustment mechanism in which rotation of the horizontal roller simultaneously activated the vertical roller, allowing accurate setting of the desired scale values (Fig. 4). The horizontal roller was designed with 20 indexed settings, labeled from 0 to 9 in 0.5-unit intervals, while the vertical roller featured three separate notch positions. A full rotation of the horizontal roller shifted the vertical roller forward by one scale level, advancing it from position 0 to position 1.

To evaluate seed flow characteristics—namely seed rate and uniformity—collection containers were placed beneath the outlets of the conveyor belt-type metering device. In each test run, seeds were released from the metering unit for a 60-second period using the weighing setup illustrated in Fig. 3.

Each experimental condition was tested in three replications, and all trials were carried out in a randomized sequence. The seed metering device operated at a forward speed of 1.0 m s^−^^1^, and a range of seed rate settings was examined to determine the resulting seed output. Seed quantities were measured using a digital scale with a resolution of 0.01 g, and all measurements were documented in Microsoft Excel.

For the evaluation of flow uniformity, the seed metering device was tested according to the factors specified in the experimental design. The resulting data were analyzed using coefficient of variation (CV, %) values, following the procedure outlined by Önal (2017), and the outcomes are summarized in Table 2.

**Table 2:** Subjective assessment for seed flow uniformity.

CV (%)	Assessment
<1	Very good
1–2	Good
2–3	Moderate
3–4	Sufficient

In-row seed distribution uniformity was assessed using the variation factor (V_f_) and the goodness criterion (*λ*), both derived through computer-aided classification. The V_f_ value reflects how closely the observed distribution follows a Poisson pattern, while *λ* indicates the proportion of segments that contain one, two, and three seeds or plants.

In-row seed spacing was quantified using the V_f_ values obtained from the experiments, and the uniformity of this spacing was further interpreted through a goodness criterion. The calculated V_f_ and *λ* values are summarized in Table 3. The equations used to determine the variation factor (V_f_) and the variance of seed distribution (S^2^) are given in Eqs. (1) and (2), following the approach described by Griepentrog (1994). The equation of the goodness criterion is presented as Eq. (3).

(1) \begin{eqnarray*}{V}_{f}= \frac{{S}^{2}}{\mu } \end{eqnarray*}

(2) \begin{eqnarray*}{S}^{2}= \frac{\sum _{i=1}^{n}{x}_{i}^{2}{f}_{i}-({ \left( {x}_{i}{f}_{i} \right) }^{2}/n)}{n-1} \end{eqnarray*}

**Table 3:** Qualitative evaluation for V_f_ and *λ*.

V_*f*_	Seeding character	*λ*	Seeding quality
		≥72	Very good
>1.1	Undesired misses and multiples	72-≥65	Good
0.9-1.1	Poisson distribution	65 - ≥55	Moderate
<0.9	Precision seeding	<55	Insufficient

where

µ : the average number of seeds per segment

x_i_: the expected number of seeds or plants in the segment

f_i_: segment ratio that is the percentage of the segments with different number of seeds

n*:* total sample number

(3) \begin{eqnarray*}\lambda =f(1)+f \left( 2 \right) +f(3)\end{eqnarray*}

where

*f* (1): the proportion of segments containing 1 seed or plant in total number of segments

*f* (2): the proportion of segments containing 2 seeds or plants in total number segments

*f* (3): the proportion of segments containing 3 seeds or plants in total number segments

For calculating the goodness criterion, the average number of seeds or plants per segment (µ ) was taken as 2, and the segment length (a) was obtained from Eq. (4) according to the method described by Önal (2017).

(4) \begin{eqnarray*}a= \frac{100\mu \sigma }{bN} \end{eqnarray*}

where

*σ*: thousand seed mass (g/1000 seeds)

b: row spacing (cm)

N: seed rate (kg ha^−1^)

A three-variable Central Composite Design (CCD), belonging to the response surface methodology framework, was employed to evaluate the metering unit’s performance in terms of both flow uniformity and in-row seed distribution. In this study, flow uniformity and the two-distribution metrics, the variation factor and goodness criterion were taken as the dependent variables. The corresponding influencing factors, designated as independent variables, were forward speed (X_1_), seed rate (X_2_), and seed falling angle (X_3_).

In a three-variable CCD, each independent variable is evaluated at five coded levels: ±1.682 for the axial points, ±1 for the factorial points, and 0 for the center point (Box & Draper, 1987). The coded and corresponding actual (uncoded) values used in this study are presented in Table 4. The metering unit was tested under five settings of forward speed, seed rate, and seed falling angle for black cumin seeding. Following CCD methodology, all experimental combinations were performed in triplicate to assess both flow uniformity and in-row seed distribution performance.

Microsoft Excel and Minitab Release 18.0 Trial Version were used for data processing and model construction, whereas Maple 17.0 Trial Version (Single User Profile) was employed to calculate the optimal values of the independent variables based on the cubic model equation.

## Results and Discussion

### Seed rate flow test results

**Figure 5:**
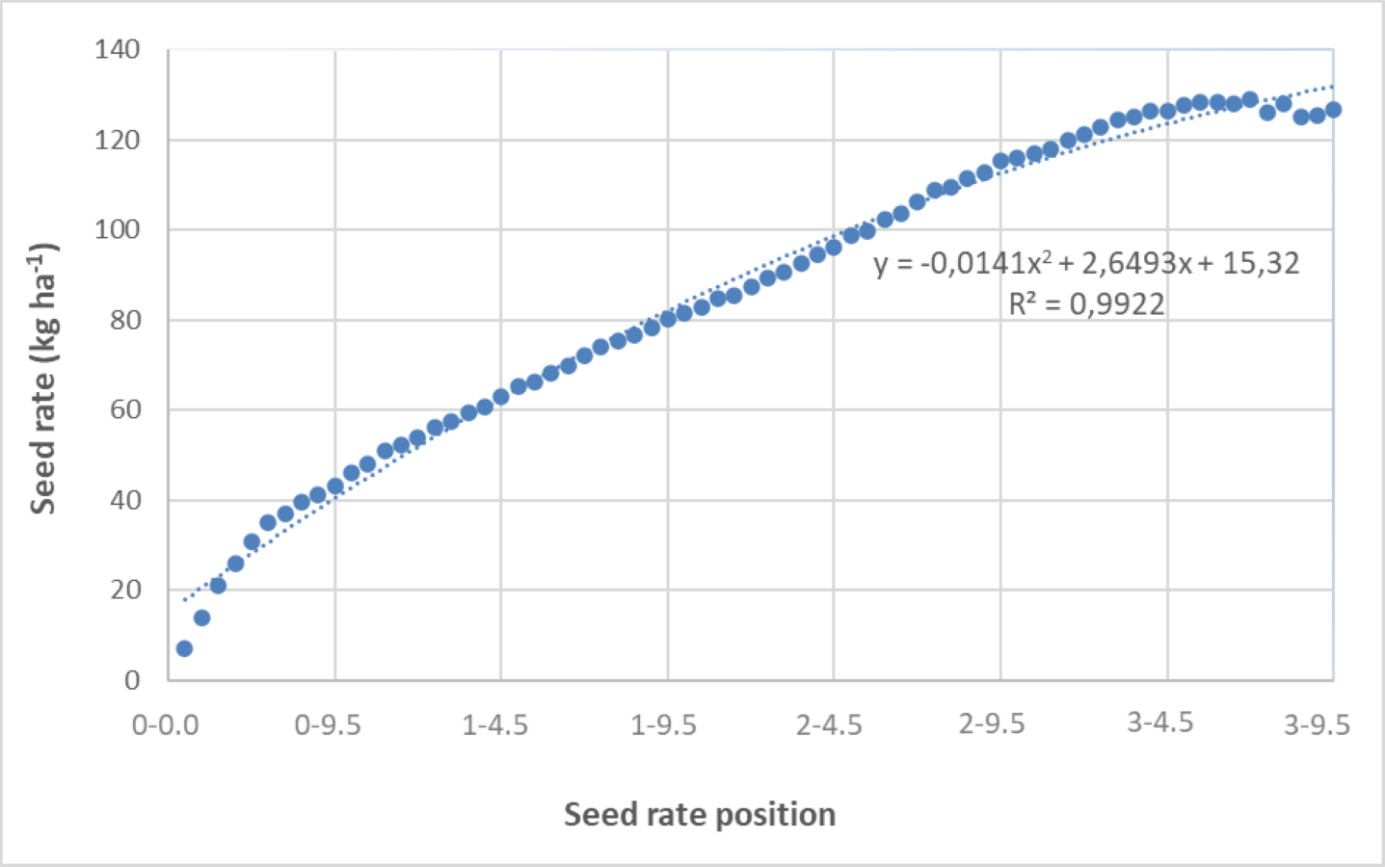
Variation of seed rate based on the position of seed rate adjustment mechanism.

Flow tests were performed at different positions of the seed rate-adjustment mechanism to find the seed rate values of black cumin at a forward speed of 1.0 ms^−1^ for 60 s, with three replications. In accordance with ISO 7256-2 (1984), which specifies that “five runs lasting 30 s shall be made for each test” and that the test rig shall provide an overall effective length of 30 m, each trial in this study was conducted for 60 s to obtain a sufficient sample size and stable seed flow. This duration ensured reliable and repeatable data for statistical analysis. Table 5 and Fig. 5 present the measured seed rate flow values produced by the seed metering unit.

**Table 4:** Independent variables and their coded levels applied for black cumin seeding.

Independent variable	Step value	Coded and uncoded (reel) values				
		−1.682	−1	0	1	1.682
Forward speed (X_1_, ms^−1^)	0.6	1.01	1.4	2.0	2.6	3.01
Seed rate (X_2_, kg ha^−1^)	5.0	6.6	10	15	20	23.4
Seed falling angle (X_3_, °)	20	1.36	15	35	55	68.64

**Table 5:** Results of seed rates based on weighing tests.

Seed rate setting main position (vertical roller)	Seed rate setting precise position (horizontal roller)	Seed weight (g)			Average (g)	Seed rate (kg ha^−1^)	CV (%)
		Rep# 1	Rep# 2	Rep# 3			
	5.0	4.34	4.11	4.22	4.22	7.0	2.72
	6.0	13.78	13.39	13.56	13.58	22.6	1.44
0	7.0	18.45	18.23	18.83	18.50	30.8	1.64
	8.0	22.00	22.30	22.34	22.21	37.0	0.84
	7.0	24.89	24.57	24.96	24.81	41.3	0.84
	0.0	30.09	30.49	29.17	29.92	49.9	2.26
	1.0	30.58	30.55	30.59	30.57	51.0	0.07
	2.0	32.54	32.13	32.48	32.38	54.0	0.68
	3.0	34.47	34.65	34.10	34.41	57.3	0.82
1	4.0	36.65	36.06	36.32	36.34	60.6	0.81
	5.0	39.23	38.92	39.07	39.07	65.1	0.40
	6.0	41.17	40.79	40.60	40.85	68.1	0.71
	7.0	43.51	43.15	43.27	43.31	72.2	0.42
	8.0	44.76	45.80	44.87	45.14	75.2	1.27
	9.0	46.59	47.39	47.12	47.03	78.4	0.87
	0.0	48.20	47.92	50.41	48.84	81.4	2.79
	1.0	51.27	49.92	51.60	50.93	84.9	1.75
	2.0	52.03	52.34	52.77	52.38	87.3	0.71
	3.0	54.08	53.99	55.16	54.41	90.7	1.20
2	4.0	55.78	56.93	57.40	56.70	94.5	1.47
	5.0	58.77	59.50	59.37	59.21	98.7	0.66
	6.0	61.30	61.04	61.73	61.36	102.3	0.57
	7.0	63.47	63.68	64.23	63.79	106.3	0.62
	8.0	65.79	65.57	65.83	65.73	109.6	0.21
	9.0	67.62	67.58	67.50	67.57	112.6	0.09
	0.0	71.32	71.00	71.60	71.31	118.8	0.42
	1.0	71.86	72.85	72.47	72.39	120.7	0.69
	2.0	73.43	73.54	71.17	72.71	121.2	1.84
	3.0	75.04	74.07	74.81	74.64	124.4	0.68
3	4.0	76.27	75.45	75.64	75.79	126.3	0.57
	5.0	76.62	77.40	76.13	76.72	127.9	0.83
	6.0	77.65	76.78	76.85	77.09	128.5	0.63
	7.0	77.36	77.52	77.51	77.46	129.1	0.12
	8.0	77.41	77.31	75.73	76.82	128.0	1.23
	9.0	74.83	74.90	76.12	75.28	125.5	0.96

Because seed flow initiated at the 0–5.00 position due to the low flowability and irregular shape of black cumin seeds, measurements were conducted in 1.0 increments starting from that point. This behavior indicates that a short initial accumulation zone is required before achieving a stable seed stream, emphasizing the importance of calibrating the outlet position and feeding rate during metering unit adjustment.

As shown in Table 5 and Fig. 5, seed rate values exhibited consistently with higher scale settings. The relationship between scale position and seed rate followed a polynomial pattern, and the resulting model achieved a coefficient of determination (R^2^) of 99.22%.

The seed rates ranged from 7.0 kg ha^−^^1^ to 125.5 kg ha^−^^1^, and the low CV values observed among replications indicate consistent and precise seed delivery. This result suggests that the metering unit maintained a uniform seed flow under the tested conditions, minimizing variation between successive trials. Considering that the recommended seed rate for black cumin is between 10 kg ha^−^^1^ and 20 kg ha^−^^1^, the results confirm that the system performed within agronomically acceptable limits. The observed consistency among replications also implies that the calibration of the metering unit and the selected operational parameters were effective in achieving reliable and uniform seed distribution.

The metering unit’s stable performance across a wide range of operating conditions suggests its potential to meet different field operation demands (Singh, Singh & Saraswat, 2005; Önal, 2017). The uniformly low CV values obtained throughout the tests demonstrate consistent seed flow, which is critical for achieving even crop emergence (Önal, 2017). These outcomes show that the system can deliver precision seeding not only within standard application ranges but also at slightly elevated seed rates, offering additional flexibility to adapt to various agronomic conditions or cultivar-specific requirements.

### Experimental results for flow uniformity

Flow uniformity of black cumin seeds was assessed through experiments structured according to CCD priciples, in which five levels of forward speed, seed rate, and seed falling angle were tested. Each experimental setting was repeated three times. The independent variables were coded at the CCD levels of −1.682, −1, 0, +1, and +1.682, with step intervals defined separately for each factor.

Table 6 includes the CCD experimental layout together with the coded and uncoded values of the independent variables, whereas Table 7 summarizes the flow uniformity results obtained from the CCD experiments.

**Table 6:** Experimental design that includes coded and uncoded values of independent variables.

Experiment number	Forward speed (X_1_)		Seed rate (X_2_)		Seed falling angle (X_3_)	
	Coded	Uncoded (ms^−1^)	Coded	Uncoded (kg ha^−1^)	Coded	Uncoded (°)
1	−1	1.4	−1	10	−1	15
2	−1	1.4	1	20	−1	15
3	1	2.6	−1	10	−1	15
4	1	2.6	1	20	−1	15
5	−1	1.4	−1	10	1	55
6	−1	1.4	1	20	1	55
7	1	2.6	−1	10	1	55
8	1	2.6	1	20	1	55
9	−1.682	1.01	0	15	0	35
10	1.682	3.01	0	15	0	35
11	0	2.0	−1.682	6.6	0	35
12	0	2.0	1.682	23.4	0	35
13	0	2.0	0	15	−1.682	1.36
14	0	2.0	0	15	1.682	68.64
15	0	2.0	0	15	0	35
16	0	2.0	0	15	0	35
17	0	2.0	0	15	0	35
18	0	2.0	0	15	0	35
19	0	2.0	0	15	0	35
20	0	2.0	0	15	0	35

**Table 7:** Flow uniformity performance results of the seed metering unit.

	Independent variables			Seed weight (g)			Average (g)	CV${}_{fu}^{\ast }$, % (evaluation)	Standard deviation
Run #	X_1_	X_2_	X_3_	Rep #1	Rep #2	Rep #3			
1	−1	−1	−1	8.92	9.45	9.32	9.23	2.99 (moderate)	0.28
2	−1	1	−1	18.02	17.90	18.12	18.01	0.61 (very good)	0.11
3	1	−1	−1	14.87	15.72	15.23	15.27	2.79 (moderate)	0.43
4	1	1	−1	30.74	31.08	30.89	30.90	0.55 (very good)	0.17
5	−1	−1	1	10.02	11.24	10.28	10.51	6.11 (insufficient)	0.64
6	−1	1	1	18.21	18.42	18.64	18.42	1.17 (good)	0.22
7	1	−1	1	15.80	16.59	15.44	15.94	3.69 (sufficient)	0.59
8	1	1	1	31.04	30.68	30.91	30.88	0.59 (very good)	0.18
9	−1.682	0	0	9.44	9.88	9.46	9.59	2.58 (moderate)	0.25
10	1.682	0	0	29.59	29.42	29.82	29.61	0.68 (very good)	0.20
11	0	−1.682	0	8.62	9.31	9.09	9.01	3.91 (sufficient)	0.35
12	0	1.682	0	29.86	30.13	30.03	30.01	0.45 (very good)	0.14
13	0	0	−1.682	18.52	18.16	18.20	18.29	1.08 (good)	0.20
14	0	0	1.682	1.26	1.88	1.83	1.66	20.79 (insufficient)	0.34
15	0	0	0	18.21	17.93	18.22	18.12	0.91 (very good)	0.16
16	0	0	0	18.04	18.27	17.84	18.05	1.19 (good)	0.22
17	0	0	0	17.81	18.12	17.90	17.94	0.89 (very good)	0.16
18	0	0	0	17.92	18.09	18.56	18.19	1.82 (good)	0.33
19	0	0	0	18.20	17.64	18.14	17.99	1.71 (good)	0.31
20	0	0	0	18.02	18.07	18.37	18.15	1.04 (good)	0.19

Each experimental run was conducted with a distinct combination of factor settings. For example, in the third run, the forward speed, seed rate, and seed falling angle were set to 2.6 m s^−^^1^, 10 kg ha^−^^1^, and 15°, which corresponded to the coded levels of +1, –1, and –1. Flow uniformity for all trials was evaluated using the CV% values summarized in Table 2.

As seen in Table 7, the mean flow rates obtained from the weighing tests ranged between 1.66 and 30.90 g for black cumin seeds. Although these values shifted according to the test conditions, the minimum and maximum CV_fu_ values across replications were 0.45% and 20.79%. Moreover, the standard deviations calculated for all experimental settings were relatively low, indicating a high level of consistency among the repeated measurements.

Evaluation of the black cumin flow uniformity results showed that the metering performance predominantly fell within the “very good” to “good” range. However, in experiments 7 and 11, the flow was classified as “sufficient”, in other words, it exhibited a borderline quality. In experiments 5 and 14, on the other hand, the flow uniformity in terms of the coefficient of variation (CV) was found to be ‘insufficient’. Based on these results, it was concluded that the flow uniformity in black cumin seeding is highly influenced by the variation in the levels of the selected parameters.

Additional support for this observation comes from experiments 6 and 13, which yielded nearly the same CV_fu_ values despite being carried out under differing factor settings.

The data in Table 7 show that flow uniformity generally improves as the applied seed rate increases. For example, at seed rates of 20 kg ha^−1^ (coded value of +1) and 23.4 kg ha^−1^ (coded value of +1) the metering unit’s flow uniformity performance was rated as “very good”, generally. However, experiment 11, which applied the lowest seed rate of 6.6 kg ha^−1^, the planter unit’s performance was rated as “sufficient”.

A previous study by Turkusay & Yazgi (2024), which used a comparable metering mechanism with sage seeds, reported different results. Their findings indicated that forward speed was the dominant factor affecting flow stability in the sage seeding. In contrast, the present study determined that, for black cumin, seed rate exerted the strongest influence on achieving consistent distribution.

Another study employed a fluted-roller type seed metering unit for coriander seeding (Varol & Degirmencioglu, 2022). In that work, the coefficient of variation (CV) for seed distribution uniformity was reported to range between 0.28% and 1.05%, varying with forward speeds of 1.0, 1.5, and 2.0 m s^−^^1^ and application rates of 15, 20, and 25 kg ha^−^^1^.

It is likely that the contrasting outcomes arise from variations in seed types or metering unit designs utilized in previous research.

### Experimental results for in-row seed distribution uniformity

The experimental arrangement presented in Table 6, which had been employed earlier to evaluate flow uniformity, was likewise adopted for the sticky belt experiments. The findings of these experiments are presented in Table 8 and were interpreted using the variation factor (V_f_) and the goodness criterion (*λ*), as explained in the Materials and Methods section. From the resulting data, the parameters S^2^, V_f_, µ , and *λ* were derived.

**Table 8:** Performance evaluation of the seed metering unit in terms of in-row seed distribution uniformity.

	Independent variables			S^2^	V_*f*_	Assessment^*^	µ	*λ*	Assessment
Run #	X_1_	X_2_	X_3_						
1	−1	−1	−1	2.67	1.33	NBD	2.52	58.33	Moderate
2	−1	1	−1	1.58	0.79	BD	2.07	75.00	Very good
3	1	−1	−1	2.97	1.48	NBD	2.80	60.00	Moderate
4	1	1	−1	1.12	0.56	BD	1.49	61.00	Moderate
5	−1	−1	1	2.47	1.24	NBD	2.49	62.33	Moderate
6	−1	1	1	1.53	0.77	BD	1.77	66.00	Good
7	1	−1	1	3.30	1.65	NBD	2.92	57.33	Moderate
8	1	1	1	1.05	0.52	BD	1.50	68.00	Good
9	−1.682	0	0	2.69	1.34	NBD	2.50	62.33	Moderate
10	1.682	0	0	1.76	0.88	BD	1.98	66.00	Good
11	0	−1.682	0	3.70	1.85	NBD	3.35	48.33	Insufficient
12	0	1.682	0	1.00	0.50	BD	1.39	61.00	Moderate
13	0	0	−1.682	1.83	0.91	PD	2.16	71.67	Good
14	0	0	1.682	1.78	0.89	BD	2.15	73.67	Very good
15	0	0	0	2.55	1.28	NBD	2.69	63.33	Moderate
16	0	0	0	2.03	1.01	PD	2.19	60.67	Moderate
17	0	0	0	2.04	1.02	PD	2.20	60.33	Moderate
18	0	0	0	2.46	1.23	NBD	2.48	62.67	Moderate
19	0	0	0	2.04	1.02	PD	2.20	66.33	Moderate
20	0	0	0	1.93	0.96	PD	2.09	58.33	Moderate

**Notes.**

* BD, Binomial Distribution, NBD, Negative Binomial Distribution, PD, Poisson Distribution

Table 8 shows that the highest V_f_ value, 1.85, occurred in experiment 11, which involved black cumin seeds at a forward speed of 2 m s^−^^1^, a seed rate of 6.6 kg ha^−^^1^, and a seed falling angle of 35°. Conversely, experiment 12 yielded the minimum V_f_ value of 0.50 under the same speed and angle conditions but at the maximum seed rate of 23.4 kg ha^−^^1^. This finding emphasized the importance of seed rate in black cumin seeding.

In experiment 11, which produced a V_f_ value of 1.85, the seed distribution conformed to a negative binomial distribution, suggesting the occurrence of both undesirable multiples and missing seeds during planting. Meanwhile, experiment 12, with a V_f_ value of 0.50, exhibited a distribution consistent with a binomial distribution, indicating that the seed placement met the criteria for precision seeding.

As presented in the results, experiment 2 produced the highest *λ* value, 75.00%, which falls within the very good classification and was obtained at a forward speed of 1.4 m s^−^^1^, a seed rate of 20 kg ha^−^^1^, and a seed dropping angle of 15°. In contrast, the lowest *λ* value of 48.33% (rated as insufficient) was recorded in experiment 11, which was carried out at 2 m s^−^^1^ with a seed rate of 0.66 kg ha^−^^1^ and a seed falling angle of 35°.

The results indicated that the µ values were typically close to 2. Only experiment 11, which exhibited insufficient seeding performance, showed a µ value of 3.35. This suggests that the number of seeds deposited onto the sticky belt was approximately 1.5 times the intended amount and occurred in an irregular manner.

The variations observed in Vf, *λ*, and µ values demonstrate that the selected independent variables significantly influenced the seeding performance of the metering unit. When changes in these variables alter the system’s behavior, it indicates that the system responds distinctly to each condition. This responsiveness suggests that the system is appropriate for developing mathematical models.

### Mathematical modelling of the performance

One of the important targets of this research was to identify which operating parameters most strongly influence the precision placement of black cumin seeds when using a conveyor belt-type metering device. For this target, cubic regression models were formulated for flow uniformity (CV_fu_) as well as in-row distribution uniformity (V_f_ and *λ*), using the experimental data summarized in Tables 7 and 8.

Comprehensive evaluations performed with the Minitab Release 18.0 Trial Version demonstrated that statistically significant cubic (3rd-degree) models were obtained for CV_fu_ and *λ* at the 99% confidence level, whereas the cubic model for V_f_was found to be significant at the 90% level. The transformation of “ln” was applied to CV_fu_ values to normalize the data distribution and reduce right skewness, while the transformation of “square root” was applied to V_f_ and *λ* values to stabilize variance and improve model fit.

Stepwise regression analysis led to the formulation of predictive models for flow uniformity (y_CVfu_), variation factor (y_Vf_), and goodness criteria (y_λ_) during black cumin seeding with a conveyor belt-type seed metering unit. The statistical results corresponding to these models, generated through Minitab, are presented in Tables 9, 10 and 11.

(5) \begin{eqnarray*}\ln \nolimits ({\mathrm{y}}_{\mathrm{CVfu}})=0.1631-0.643{\mathrm{X}}_{2}+0.296{\mathrm{X}}_{3}^{3}+0.431{\mathrm{X}}_{3}^{2}-0.145{\mathrm{X}}_{1}^{3}-0.127{\mathrm{X}}_{1}{\mathrm{X}}_{3}-0.195{\mathrm{X}}_{1}^{2}{\mathrm{X}}_{2}\nonumber\\\displaystyle  ({R}^{2}=95.34\%)\end{eqnarray*}

(6) \begin{eqnarray*}\sqrt{{y}_{Vf}}=1.046-0.262{\mathrm{X}}_{2}-0.0516{\mathrm{X}}_{3}^{2}-0.068{\mathrm{X}}_{1}{\mathrm{X}}_{2}-0.0208{\mathrm{X}}_{1}^{3}+0.072{\mathrm{X}}_{1}^{2}{\mathrm{X}}_{2}+0.0262{\mathrm{X}}_{2}^{2}\nonumber\\\displaystyle  ({R}^{2}=94.26\%)\end{eqnarray*}

(7) \begin{eqnarray*}\sqrt{{y}_{\lambda }}=7.852+0.372{X}_{2}+0.249{X}_{3}^{2}-0.225{X}_{2}^{2}+0.178{X}_{1}{X}_{2}{X}_{3}-0.141{X}_{1}{X}_{2}^{2}+0.066{X}_{1}^{2}\nonumber\\\displaystyle  -0.124{X}_{1}^{2}{X}_{2}+0.068{X}_{1}{X}_{3}-0.063{X}_{1}{X}_{2}+0.0231{X}_{1}^{3}({R}^{2}=95.96\%).\end{eqnarray*}

As indicated by the cubic models initially formulated with 19 variables, the stepwise regression analyses performed in Minitab retained six variables for CV_fu_, six for V_f_, and 10 for *λ*, respectively.

As shown in Table 9, the seed rate (X_2_) was the first and most influential variable in the CV_fu_ model, explaining 44.94% of the system variation. The subsequent variables were the seed falling angles (X_3_^3^ and X_3_^2^), which together accounted for approximately 42% of the variance. These results indicate that the seed rate and seed falling angle were the dominant factors affecting seed flow uniformity, while the forward speed had a comparatively minor influence.

**Table 9:** The Minitab-based statistical results for y_CVfu_ model.

Step no	Model predictors	Model coefficient	Standard deviation	R^**2**^ variation (%)
–	Model constant	0,163	–	–
1	X_2_	−0.643	0.704	44.94
2	X_3_^3^	0.296	0.508	71.80
3	X_3_^2^	0.431	0.342	87.47
4	X_1_^3^	−0.145	0.241	93.87
5	X_1_X_3_	−0.127	0.228	94.62
6	X_1_^2^X_2_	−0.195	0.214	95.34

**Table 10:** The Minitab-based statistical results for y_Vf_ model.

Step no	Model predictors	Model coefficient	Standard deviation	R^2^ variation (%)
–	Model constant	1.046	–	–
1	X_2_	−0.262	0.0957	78.81
2	X_3_^2^	−0.0516	0.0841	83.92
3	X_1_ X_2_	−0.068	0.0724	88.28
4	X_1_^3^	−0.0208	0.0640	91.03
5	X_1_^2^ X_2_	0.072	0.0568	93.06
6	X_2_^2^	0.0262	0.0521	94.26

**Table 11:** The Minitab-based statistical results for y_λ_ model.

Step no	Model predictors	Model coefficient	Standard deviation	R^2^ variation (%)
-	Model constant	7.852	–	–
1	X_2_	0.372	0.361	32.66
2	X_3_^2^	0.249	0.281	59.80
3	X_2_^2^	−0.225	0.196	80.73
4	X_1_X_2_ X_3_	0.178	0.160	87.48
5	X_1_ X_2_^2^	−0.141	0.141	90.43
6	X_1_^2^	0.066	0.129	92.14
7	X_1_^2^ X_2_	−0.124	0.118	93.50
8	X_1_X_3_	0.068	0.110	94.48
9	X_1_ X_2_	−0.063	0.103	95.31
10	X_1_^3^	0.0231	0.0961	95.96

As shown in Table 10, the X_2_ term, representing the seeding rate, entered the model first and alone explained 78.81% of the system. The second variable, X_3_^2^, corresponding to the seed falling angle, contributed to the system by approximately 5%. The interaction term X_1_X_2_, incorporated as the third variable in the model, accounts for approximately 4% of the system’s variability, representing the combined effects of forward speed and seed rate. Variables added in subsequent steps contribute progressively smaller improvements to the model’s prediction coefficient.

As can be seen from Table 11, the X_2_ seeding rate independent variable, which enters the model in the first position, explains 32.66% of the system on its own. Approximately 26% of the system is explained by the X_3_^2^ term which entered the model in the second positions of the seed falling angle independent variable. The third variable entered to the model was X_2_^2^, representing the quadratic term of the seeding rate. According to the analysis, seed rate emerged as the most effective independent variable in the *λ* model for black cumin seeding. In contrast, the impact of forward speed on the system was noticeably minimal compared with the other two parameters.

The developed model equations are valid only within the specified parameter ranges of the system. Beyond these limits, their predictive reliability may decrease, and results should therefore be interpreted with caution.

1.01 m s^−1^ ≤ Forward speed ≤ 3.01 m s^−1^

6.6 kg ha^−1^ ≤ Seed rate ≤ 23.4 kg ha^−1^

1.36° ≤ Seed falling angle ≤ 68.64°.

### Optimization of the in-row seed distribution uniformity performance

One of the main purposes of this study was to determine the optimal operating conditions that promote accurate and efficient seeding of black cumin. For this purpose, the model equations describing in-row seed distribution uniformity were utilized in Maple to perform the optimization process.

The roots of each equation were identified, and the optimal coded values of all independent variables were subsequently calculated.

To determine the optimal input levels for V_f_ and*λ*, the first derivatives of the model equations with respect to each independent variable were calculated, and stationary points were obtained by setting these derivatives equal to zero.

For the V_f_ model, the optimum coded levels were calculated as −1.588 for X_1_, −0.5307 for X_2_, and 0 for X_3_. Converting these coded levels to actual operating parameters yielded values of 1.05 m s^−^^1^ for forward speed, 12.35 kg ha^−^^1^ for seed rate, and a seed falling angle of 35°.

Likewise, the optimum coded levels for the *λ* model were determined as −0.7444, 1.2229, and 0.4270 for X_1_, X_2_, and X_3_, respectively. When converted to actual operating conditions, these corresponded to a forward speed of 1.55 m s^−^^1^, a seed rate of 21.1 kg ha^−^^1^, and a seed falling angle of 28.5° .

The optimum values of forward speed, seed rate and seed falling angle values were found different for both the V_f_ and *λ* models. As shown in Tables 10 and 11, the *λ* model exhibited a higher R^2^ value than the V_f_model, demonstrating its superior ability to predict variability within the system.

Accordingly, the optimal settings derived from the *λ* model are likely to be more informative and reliable than those obtained from the V_f_model.

In a study by Turkusay & Yazgi (2024) involving sage seeds, the researchers achieved V_f_ and *λ* models with similar results. In that research, the V_f_ model showed a higher R^2^ value (97.34%) than the *λ* model, which had an R^2^ of 92.27%.

Validation experiments were conducted using the optimum conditions identified for both models: 1.05 m s^−1^ forward speed, 12.35 kg ha^−1^ seed rate, and a 35° seed falling angle for the V_f_ model; and 1.55 m s^−1^, 21.1 kg ha^−1^, and 28.5° for the *λ* model.

For each optimum condition, three replications were performed, and the seed metering unit’s in-row distribution uniformity was evaluated accordingly. The findings from these validation tests are summarized in Table 12.

**Table 12:** The results of verification tests performed by using optimum conditions.

Model	Optimum conditions	µ	V_*f*_	*λ*(%)
	1.05 m s^−1^			
V_f_ model	12.35 kg ha^−1^	2.66	0.93	82.33
	35°			
	1.55 m s^−1^			
*λ* model	21.1 kg ha^−1^	1.85	0.53	91.67
	28.5°			

As shown in Table 12, seeding performed under the *λ* model’s optimal conditions yielded a more favorable seed living space than that obtained from the V_f_ model. This is because, as previously noted, the µ  value should equal or approximate the theoretical target of 2.

The variation factor value obtained from the *λ* model was found to be smaller than the V_f_ model. It may be considered that *λ* model a more suitable model based on the variation factor. Based on the results, it was determined that under the optimum conditions derived from the *λ* model, the sowing of black cumin seeds could be performed with the characteristics of “precision seeding”.

Moreover, experiments carried out under optimum conditions demonstrated that the *λ* model provided a higher goodness criterion value than the V_f_ model, indicating that better quality seeding can be achieved under these conditions.

In the CCD-based experiments, the *λ* values varied between 48.33% and 75.00%, as shown in Table 8. In contrast, when the tests were conducted under the optimized conditions determined by the V_f_ and*λ* models, the *λ* value reached 82.33% and 91.67%, respectively as shown in Table 12. Both values were evaluated as indicating a “very good” performance level. Under optimal conditions, this improvement signifies better seeding performance, characterized by an increased frequency of single, double, and triple seed drops across all seed rows.

Under the optimal conditions determined for the *λ* model, the V_f_ values revealed a binomial seed distribution, which qualifies as precision seeding (V_f_ <0.9) as shown in Table 12. This finding suggests that the seed metering unit is capable of precision seeding of black cumin seeds when operated under suitable working conditions. Such uniform seed distribution minimizes intra-row competition among plants, promotes even seedling emergence, and contributes to improved stand establishment and yield potential. Therefore, the optimized operational settings identified in this study not only enhance mechanical performance but also have practical importance for achieving agronomically efficient seeding.

The µ  values derived from the *λ* model under optimized conditions approached the ideal theoretical target of 2, whereas previous experiments yielded values between 1.39 and 3.35, as illustrated in Table 8. Achieving a *μ* value close to 2 indicates that the number of seeds per segment closely corresponds to the ideal binomial distribution, signifying uniform in-row seed placement. This improvement is expected to minimize misses and multiple, resulting in better seeding uniformity and stand establishment in the field. Consequently, this uniformity reduces seed losses and enhances yield efficiency, demonstrating the practical benefits of the applied optimization strategy.

The higher R^2^ value of the *λ* model, compared with that of the V_f_model, demonstrates its superior predictive capability. Consequently, the µ  value generated under the *λ* model’s optimal conditions may yield better seeding performance than that obtained from the V_f_ model.

**Figure 6:**
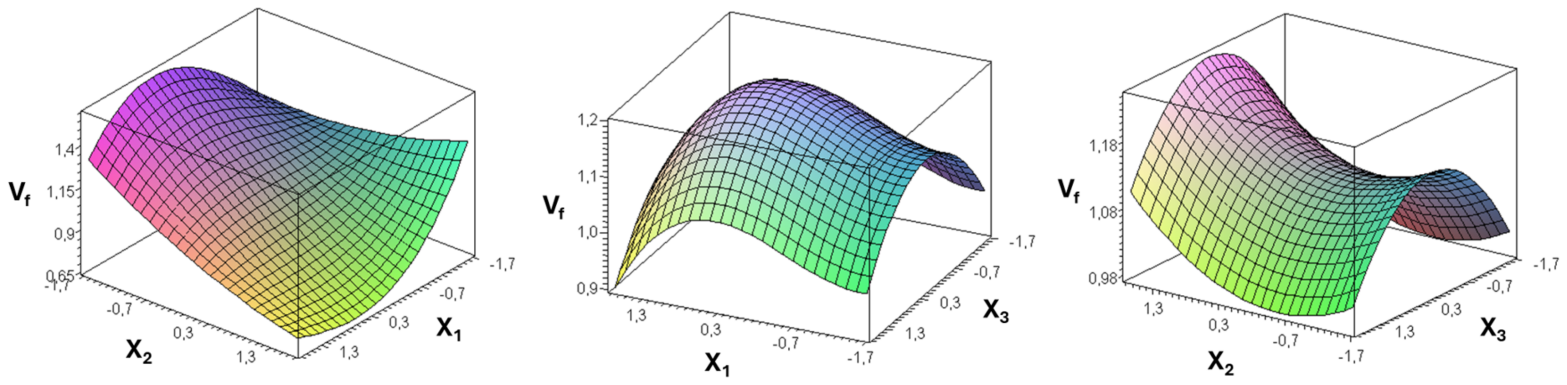
*V*_*f*_ variation as a function of coded values of forward speed (*X*_1_), seed rate (*X*_2_) and seed falling angle (*X*_3_).

**Figure 7:**
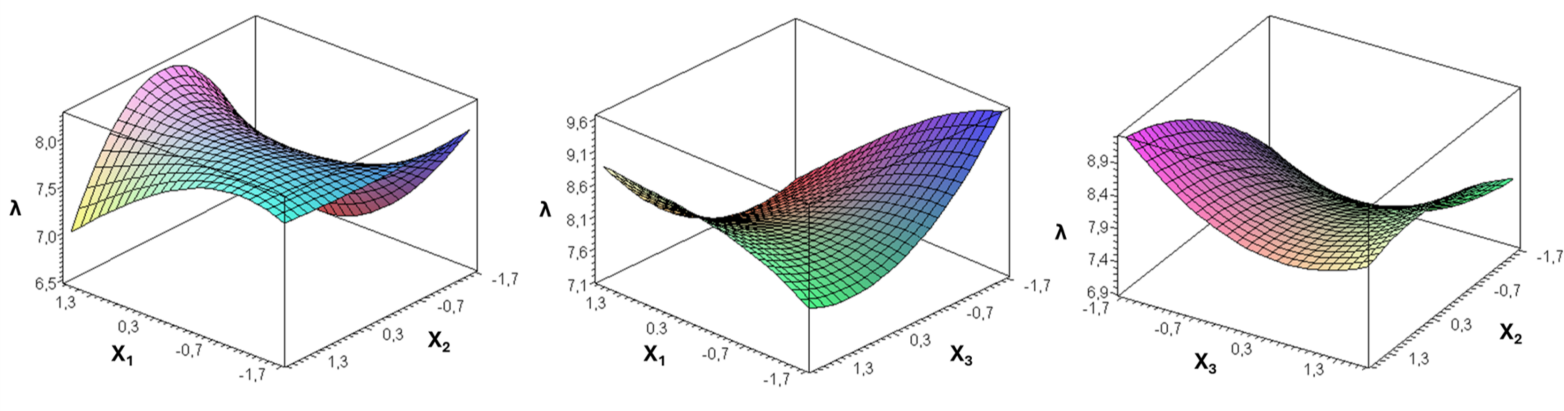
*λ* variation as a function of coded values of forward speed (*X*_1_), seed rate (*X*_2_) and seed falling angle (*X*_3_).

Figures 6 and 7 provide clear visual evidence of how the variables evaluated in this study affected the in-row distribution uniformity of black cumin seeds delivered by the metering unit.

In these figures, one independent variable was fixed at its optimal value while the other two were adjusted, enabling an assessment of their individual effects on the variation factor and the goodness criterion. The visualizations clearly reveal how forward speed, seed rate, and seed falling angle influence overall seeding performance.

Figure 6 illustrates the interactive effects of the seed rate (X_2_), forward speed (X_1_), and seed-falling angle (X_3_) on the variation factor (V_f_). In the X_2_–X_1_ interaction surface, V_f_decreased gradually as the seed rate increased up to a moderate level while the forward speed remained low, indicating smoother seed delivery; however, at higher seed rates or speeds, V_f_ rose sharply, suggesting seed accumulation and non-uniform discharge. The X_1_–X_3_ surface reveals that increasing the seed-falling angle initially reduced V_f_, implying improved drop regularity, but beyond the mid-range angle the values increased again, reflecting greater seed bounce and turbulence. A similar curvature is visible in the X_2_–X_3_ interaction, where the lowest V_f_ values—representing optimum seeding uniformity—occurred at intermediate levels of both parameters. These nonlinear patterns and turning points identify the operating zones where the metering unit transitions from uniform to irregular seed flow.

Figure 7 illustrates the interactive effects of forward speed (X_1_), seed rate (X_2_), and seed-falling angle (X_3_) on the goodness criterion (*λ*). In the X_1_–X_2_ surface, *λ* increased as both forward speed and seed rate rose from their lower levels, reaching a peak at intermediate values that represent the optimal combination for precise seed delivery. Beyond this point, further increases led to a reduction in *λ*, indicating over-metering and irregular spacing. The X_1_–X_3_ interaction surface shows that *λ* improved with moderate seed-falling angles but declined sharply at extreme angles, likely due to seed bounce and trajectory distortion within the discharge path. A similar nonlinear trend was observed in the X_2_–X_3_ plot, where high seed rates combined with large falling angles caused a noticeable drop in *λ*. Overall, these trends confirm that balanced operating settings produce the highest seeding quality, whereas extreme combinations adversely affect seed placement uniformity.

**Figure 8:**
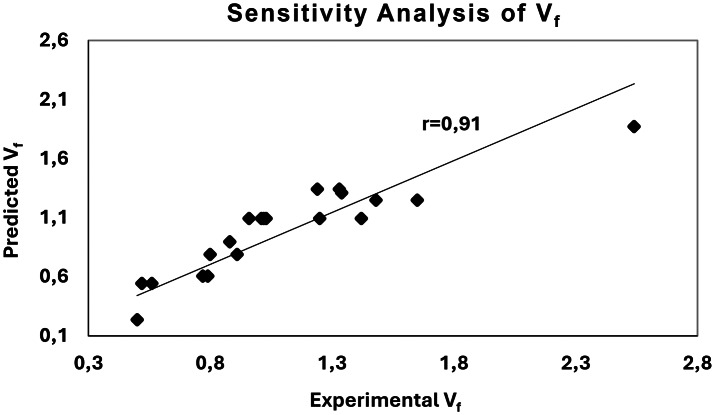
Sensitivity analysis of *V*_*f*_ model.

**Figure 9:**
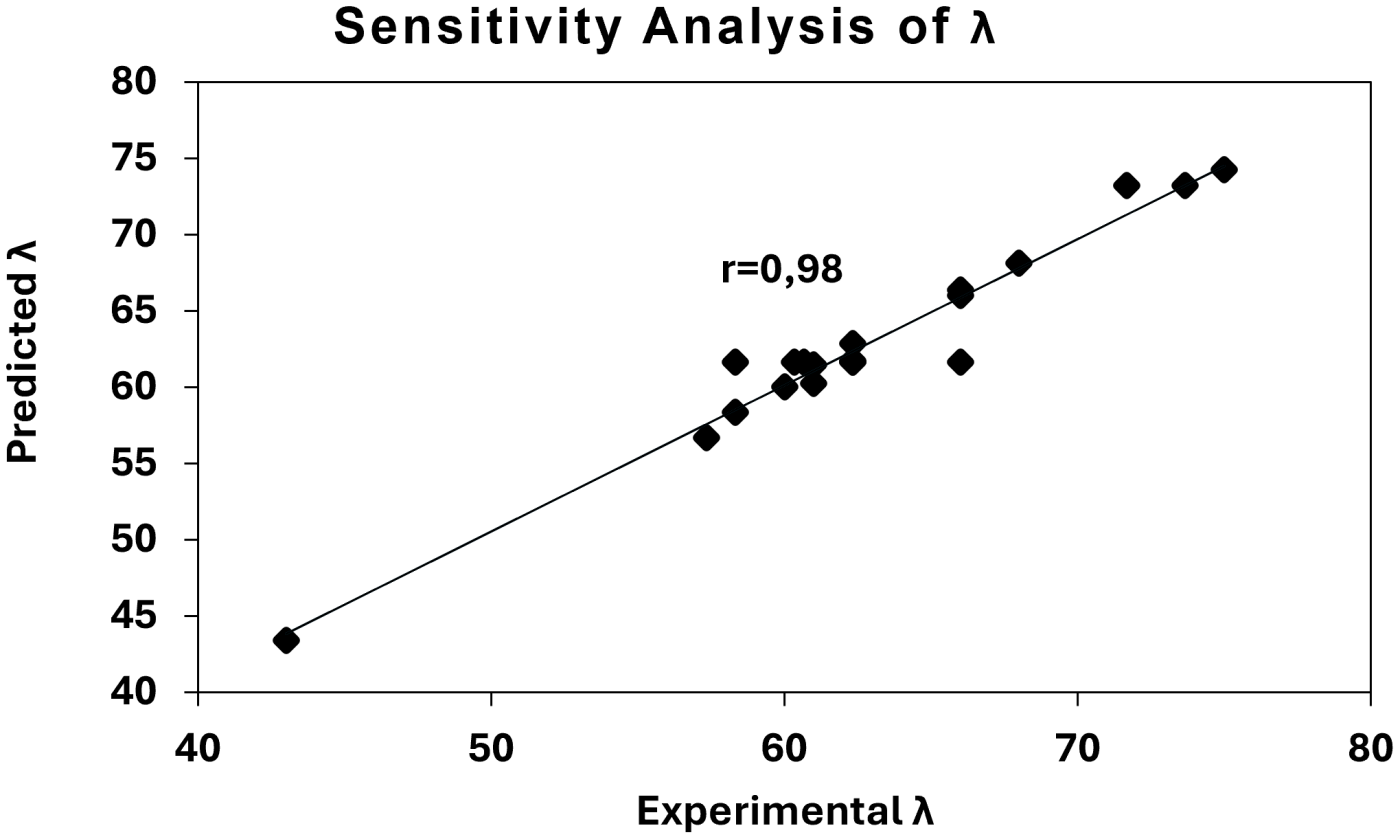
Sensitivity analysis of *λ* model.

For the sensitivity analysis, the measured data were compared with the values predicted by the model equations, as shown in Figs. 8 and 9 for the V_f_ and*λ* models, respectively.

In these graphs, the diagonal line represents a perfect correlation (r = 100%). The comparison revealed a strong consistency between the measured and predicted values, with correlation coefficients of 0.91 for the V_f_ model and 0.98 for the *λ* model. Although there is no universally defined threshold for acceptable correlation in predictive modeling, the closer the correlation coefficient is to the ideal value (*r* = 1.00), the greater the predictive accuracy achieved. These results demonstrate that the developed models can estimate seeding performance with less than 10% prediction error, confirming their suitability for precision seeding analysis and calibration purposes.

Overall, the analyses confirmed that both the V_f_ and *λ* models effectively describe the in-row seed distribution uniformity in black cumin seeding. Nevertheless, the *λ* model exhibited a higher R^2^ value than the V_f_ model, demonstrating its superior predictive capability.

These results confirm the strength of the proposed models and highlight their practical value for optimizing seed metering performance in black cumin seeding. The improved *λ* value obtained through optimization indicates enhanced planting quality, which is essential for achieving uniform crop emergence. Moreover, due to its stronger predictive capability, the *λ* model offers greater confidence in identifying the most suitable operating parameters. Overall, the modeling and optimization approach presented in this work holds considerable promise as a reference framework for precision planting applications in other small-seeded crop systems.

The performance evaluations of seeding machines conducted under laboratory conditions serve as a prediction of their field performance. By minimizing or eliminating potential errors originating from the machine before field operation, only external factors such as soil and climatic conditions remain influential under real field conditions. Therefore, a decrease in machine performance in the field, because of these external factors, is an expected outcome.

## Conclusions

Employing a conveyor belt–type seed metering mechanism in conjunction with advanced modeling and optimization methods was shown to be effective in ensuring accurate in-row seed placement for black cumin. The V_f_ and *λ* models exhibited strong predictive performance and were successfully validated under controlled experimental conditions. Their capacity to sustain uniform seed distribution under different operating settings underscores the suitability of this approach for precision seeding applications. These results support the ongoing development of precision agriculture technologies and provide practical insights for enhancing seeding efficiency and crop establishment in small-seeded crops.

The experiments in this study were conducted under strictly controlled laboratory conditions, which may not entirely reflect the variability encountered in actual field environments. To determine how well the laboratory-derived findings translate to practical field applications, future work will include field-based trials. Factors such as soil heterogeneity, vibration, seedbed roughness, and machine–soil interaction can influence the seeding performance in practice. Therefore, future studies will focus on validating the models under real field conditions, investigating the impact of external parameters, and integrating sensor-based feedback or artificial intelligence (AI)-assisted systems for real-time calibration and performance monitoring.

Although the conveyor belt–type metering unit is commonly marketed as a fertilizer attachment for seeders, it is believed that, with slight structural adjustments enabling integration into a main frame, the system could also function effectively as a standalone seeding mechanism.

This study on black cumin seeding is anticipated to provide meaningful guidance for farmers working to improve crop establishment, for manufacturers developing higher-precision seeding equipment, and for researchers advancing smart agricultural technologies.

##  Supplemental Information

10.7717/peerj.20755/supp-1 Supplemental Information 1 Sticky belt experiments raw data

## Additional Information and Declarations

### Data Availability

The following information was supplied regarding data availability: The raw measurements of sticky belt experiments are available in the Supplementary File.
